# Prevalence of age-related macular degeneration among the elderly 

**Published:** 2015

**Authors:** Seyed Ahmad Rasoulinejad, Amin Zarghami, Seyed Reza Hosseini, Neda Rajaee, Seyed Elahe Rasoulinejad, Ebrahim Mikaniki

**Affiliations:** 1 Department of Ophthalmology, Ayatollah Rouhani Hospital, Babol University of Medical Sciences, Babol, Iran.; 2 Student Research Committee, Babol University of Medical Sciences, Babol, Iran.; 3 Social Determinants of Health (SDH) Research Center, Babol University of Medical Sciences, Babol, Iran.; 4 Babol University of Medical Sciences, Babol, Iran.

**Keywords:** Aged, Smoker, Macular Degeneration, Risk factors

## Abstract

**Background:**

Age-related macular degeneration (AMD) is the leading cause of visual impairment and blindness in elderly population in the developing countries. Previous epidemiological studies revealed various potential modifiable risk factors for this disease. The purpose of this study was to evaluate the prevalence of AMD among elderly living in Babol, North of Iran.

**Methods:**

The study population of this cross-sectional study came from the Amirkola Health and Ageing Project (AHAP), the first comprehensive cohort study of the health of people aged 60 years and over in Amirkola, North of Iran. The prevalence of AMD was estimated and its risk was determined using logistic regression analysis (LRA) with regard to variables such as smoking, hyperlipidemia, hypertension and diabetes.

**Results:**

Five hundred and five participants with mean age of 71.55±5.9 (ranged 60-89) years entered the study. The prevalence of AMD was 17.6%. There was a significant association between AMD and smoking (P<0.001) but no association was seen with AMD and age, level of education, history of hyperlipidemia, hypertension and diabetes. Multiple LRAs revealed that smoking increased AMD by odds ratio of 5.03 (95% confidence interval 2.47-10.23 p<0.001) as compared to nonsmokers

**Conclusion:**

According to our findings, the prevalence of AMD was relatively high and smoking increased the risk of AMD in the elderly population.

Age-related macular degeneration (AMD) is the leading cause of visual impairment and blindness in elderly population in developing countries (1). Previous systematic review studies revealed that age-specific estimates revealed a prevalence of early AMD which was more common in European ancestry (%8.8) (2) than Asians (%6.8) (3) but this discrepancy was not seen in late AMD. Recent reports have revealed that AMD in any age is more common in European countries than Asians and Africans. As the prevalence of the disease is likely to increase in future decades as the global population exponential ageing pattern, the global burden of the AMD was estimated to reach the projected number of 196 million people in 2020 and 288 million in 2040 (4). It is hypothesized that a dysregulation in the activation of alternative pathway of complement system leads to retinal damage resulting in AMD (5-6). As there is no effective treatment for all types of AMD, identifying modifiable risk factors is of great importance (7). Numerous epidemiological studies revealed various potential risk factors for AMD. 

They were classified in different major categories including: lifestyle, genetics, nutritional, environmental and demographics. However, the strength and reliability of evidence in literature is still the matter of controversy. Among them, genetic factors, increasing age, current smokers, history of cataract surgery and family history of AMD have been documented with strong association with AMD. Risk factors such as higher body mass index, history of cardiovascular disease, hypertension, and gender, ethnicity, diabetes mellitus, alcohol use and serum lipid profile were characterized with moderation and inconsistent with AMD in some studies (8-12). Smoking has been identified as the most consistent and major modifiable risk factor for AMD (13-15).

 The growing trend of population ageing will increase the burden of AMD-related ocular morbidity which resulted in dominant impairment in quality of life, emotional management and functional independence (16-18). Therefore, realizing the burden of the disease and prevalence of the associated risk factors is necessary for making program and policy. Although there have been several population-based studies of AMD world-wide; but there was no data from elderly Iranian. On the other hand, scarce information is available about the health of older people in Iran. We aimed to determine the prevalence of AMD and assess the frequency of several AMD risk factors in an Iranian elderly population. This study was first ophthalmologic report of the Amirkola Health and Ageing Project (AHAP).

## Methods

This cross-sectional study was a part of the Amirkola Health and Ageing Project (AHAP) which is the first comprehensive cohort study of the health of people aged over 60 years in Iran who are living in a town in southern coast of Caspian Sea, North of Iran. The details of the original survey profile were described elsewhere (19). The eligible residents of Amirkola City (sixty and over) were invited to the study. After informing the participants about the objectives of the study, a written informed consent was taken from them. The project's protocol was previously approved by the Ethics committee Board of Babol University of Medical Sciences, Babol, Iran. We adhered all the stages of the study to the principles determined in Declaration of Helsinki for research. 

**Patient selection and data collection:** Inclusion criteria were individuals aged 60 and over. Smokers were attributed to those participants smoking more than 7 cigarettes per week (1 per day in average). Smoking pack years were calculated by the average number of cigarettes smoked by a participant per day divided by 20 and multiplied by the total number of years smoked. All the comprehensive ocular assessments were performed by two trained ophthalmologists based on the standard protocols. The ophthalmic examination included: visual acuity by Snellen eye charts, measurement of intraocular pressure, slit lamp evaluation and posterior pole examination by non-contact lens. During the examination, the pupils were dilated with tropicamide 1% eye drops. For the diagnosis of AMD, we used fluorecein angiography. The dominantly affected eye was used for analysis and those participants with gradable eyes in examination were considered for the estimation of the prevalence of AMD. The protocol utilized in this study follows closely the International AMD classiﬁcation and grading system (20). Structural questionnaire was designed and filled out to retrieve all the demographic status and information regarding ophthalmologic and medical history including smoking habit, level of education, vascular risk factors and others. The medical history of hypertension, diabetes and hyperlipidemia was considered based on the history of being under treatment for these medical conditions. Exclusion criteria were the non-smokers and passive smokers, and those with poor vision during examination. The ophthalmic exam was not fully accomplished for them and other ocular comorbidities at the time of the study. 

**Data analysis:** The variables analyzed in this study were defined and categorized as follows. Statistical analysis was performed using SPSS Version 18 (Chicago, USA). Educational status was divided into 3 groups: illiterate, secondary high school graduate, and higher. Smoking status among current smokers was categorized based on the smoking pack year as follows: less than 20, 20-40 and more than 40. When the data from a variable were presented as mean (SD), an independent samples t-test was used. Chi-square test was used when the data from variables presented as n (%) such as: demographic groups and medical history of hyperlipidemia, hypertension, diabetes. The first category among the categories was selected as a reference in logistic regression analysis. 

Adjusting variables were smoking, diabetes, hyperlipidemia and hypertension. The associated risk was calculated by crude and adjusted odds ratio with 95% confidence interval. A p-value less than 0.05 was considered statistically significant. 

## Results

Of the 1616 participants in the cohort setting, 505(57.4% males, 42.6% females) non-institutionalized residents met the inclusion criteria and had a gradable fundus photograph from at least one eye. The mean age of the participants was 71.55±5.9 with the 60-89 range of age. The crude prevalence of AMD in our study was (89/505) 17.6%. The mean number of cigarette smoking among the population of smokers was 35.64±28.83. 

The comparison between demographic and medical characteristic of participants with and without AMD is demonstrated in table 1. There was significant difference between AMD with smoking (p<0.001) but there was no association between AMD and other variables including: age, level of education, diabetes mellitus, hyperlipidemia and hypertension. 

**Table 1:** Comparison of Baseline Characteristics between Participants with No Age-related Macular Degeneration and Age-related Macular Degeneration in an elderly population of Amirkola (n=505)

**Characteristics**	**No Age-related** **(N= 416)**	**Age-related** **(N=89)**	**P value**
Age (Mean)	71.6±5.87	71.34±6.07	0.70
**Sex** Male Female	235(81%) 181(84.2%)	55(19%) 34(15.8%)	0.35
**Level of education** Illiterate Middle school Diploma and higher	7(70%) 332(83.2%) 77(80.2%)	3(30%) 67(16.8%) 19(19.8%)	0.45
**History of Hyperlipidemia** No Yes	353(82.3%) 63(82.9%)	76(17.7%) 13(17.1%)	0.89
**History of Hypertension** No Yes	289(83.5%) 127(79.9%)	57(16.5%) 32(20.1%)	0.31
**History of Diabetes** No Yes	328(82.8%) 88(80.7%)	68(17.2%) 21(19.3%)	0.61
**Cigarette smoking** No Yes	313(87.4%) 103(70.1%)	45(12.6%) 44(29.7%)	<0.001

As described in table 2, logistic regression analysis was performed to assess the risk factors associated with AMD among the study population by the rejection of confounding factors. When adjusted for possible confounders, it demonstrated that men who smoked had a higher risk of AMD of (multivariate adjusted OR=5.03; 95% CI, 2.47-10.23); P<0.001) compared with those that never smoked. Also, men who were nearly 2 times more likely to have AMD (multivariate adjusted OR=2.23; 95% CI, 1.09-4.57); P=0.02) than women. There is no association between AMD and other risk factors e.g. diabetes, hyperlipidemia and hypertension in our study population (table2).

**Table2:** Multivariate logistic regression analysis for determining the predictors of AMD in an elderly population of Amirkola (n=505)

**Variables**	**Crude OR** **(95%CI)**	**P value**	**Adjusted Odds ratio** **(95% CI)**	**P value**
Sex (Male)	0.80(0.50-1.28)	0.35	2.23(1.09-4.57)	0.028
History of Diabetes	1.15(0.66-1.98)	0.61	1.12(0.63-2.005)	0.69
History of Hyperlipidemia	0.95(0.50-1.82)	0.89	0.94(0.44-1.98)	0.87
History of Hypertension	1.27(0.79-2.06)	0.31	1.29(0.78-2.12)	0.31
Smoking	2.93(1.83-4.69)	<0.001	5.03(2.47-10.23)	<0.001

## Discussion

This cross-sectional study indicated the prevalence of AMD among elderly smokers from the AHAP study, a population-based cohort study in North of Iran. The overall prevalence of AMD in our study population among the 60-90 years old residents of Amirkola region was 17.6%. There was no significant difference between the prevalence of AMD with age, level of education and the history of diabetes, hyperlipidemia and hypertension. Prevalence was strongly associated with smoking.

Population-based studies all over the world documented various prevalence from 3.5% in Tromsø Eye Study in Norway (21), 12.3% from Rotterdam Study in the Netherlands (22) and 9.8% from the Blue Mountains Eye Study in Australia (23). Data from Asian studies are mostly from eastern countries and reports from western Asian countries are scarce. The overall AMD prevalence of Beijing Eye Study was 5.4% (24), the Andhra Pradesh Eye Disease Study was 10.7% (25), The Singapore Malay Eye Study was 9.9% (26), The Hisayama Study in Japan was 13.5% (27). Also, in an interesting study, Klein et al. described the prevalence of AMD in 4 racial/ethnic groups among 6170 (45 to 85 years old) subjects selected from 6 United States communities. The prevalence rates of AMD were 2.4% (Black), 4.2% (Hispanic), 4.6% (Chinese) and 5.4% (White) (28). It was previously reported that the prevalence of late AMD in those aged 40 to 79 years was comparable between the Asian and white population (29). 

Besides the genotypic differences, these comparisons could be attributed to different levels of quality of life and the higher rate of elderly population among developed western countries.

There are various determining risk factors in the literature that were associated with AMD among elderly populations (30). Our findings revealed that the frequency of AMD was strongly associated with smoking. The role of smoking on AMD occurrence was previously proven in various studies (31). 

Christen et al. indicated that current smokers of 20 or more cigarettes per day, compared with non-smokers, had an increased 2.46 times[95%CI:1.60-3.79] risk of AMD and past smokers had a modest elevation at risk of AMD (32). Their findings were consistent in a similar study among women (14). Also, pooled data from the racially similar communities [Beaver Dam Eye Study, Rotterdam Study, and Blue Mountains Eye Study] documented consistent evidence that smoking is the principal preventable exposure associated with AMD (33). Smoking seemed to affect the eyes through multiple mechanisms which is mainly having toxic effects on the retina. This dominant issue based on evidence-based reports worldwide underline the lack of awareness about the risks of developing eye disease due to smoking among both the healthcare professionals and the general public (7).

According to multivariate analysis, in our population men were nearly 2 times more likely to have AMD. Unfortunately, all smokers in our study population were men which may affect the statistical power and the weak association for of this variable. Although the substantial higher smoking rate in Asian men than women is well-known (34) but the main reason for this discrepancy here, is because of the culture and religious circumstances in our region in which smoking among women is considered beyond normal in those ages. 

The major limitation of the study is the relatively small number of participants in our study population. In addition, because the AHAP cohort sampling scheme was not designed to be statistically representative of the Iranian population, thus the estimation of the prevalence of AMD is not directly generalizable to the Iranian population. On the other hand, we did not include institutionalized individuals, and we excluded participants without any gradable fundus photographs which may cause the underestimation of AMD prevalence. 

Although we knew that the Iranian population consists of different ethnic groups and the probable genetical variations could affect the prevalence in different areas.

**Conclusion** **:** For the first time, our study documented the AMD prevalence in such population in Iran. Higher prevalence and increased risk of AMD seen among smokers underlined the need for future studies on this cohort to provide valuable data on AMD and the possible interaction between the epidemiological characteristics of this region. AMD is considered as the most common etiology of severe visual impairment among the elderly, but the treatment approach is still the matter of controversy, thus, reducing the risk of this disease via preventive strategies seemed to be another available choice.

## Notes

**Funding: **This study has been funded by the Vice-Chancellery for Research and Technology of Babol University of Medical Sciences.
